# Steryl Sinapate as a New Antioxidant to Improve Rapeseed Oil Quality during Accelerated Shelf Life

**DOI:** 10.3390/ma14113092

**Published:** 2021-06-04

**Authors:** Dobrochna Rabiej-Kozioł, Marek P. Krzemiński, Aleksandra Szydłowska-Czerniak

**Affiliations:** Faculty of Chemistry, Nicolaus Copernicus University in Toruń, Gagarina 7, 87-100 Toruń, Poland; d.rabiej@umk.pl (D.R.-K.); mkrzem@umk.pl (M.P.K.)

**Keywords:** β-sitosteryl sinapate, rapeseed oil, antioxidant activity, oxidative degradation, fluorescence spectroscopy

## Abstract

In recent years, steryl esters have become an attractive for the cosmetic, pharmaceutical, and food industries. Hence, the effect of exogenous antioxidant, β-sitosteryl sinapate on oxidative stability and antioxidant activity (AA) of refined rapeseed oil was evaluated by the accelerated shelf-life test. Oxidative parameters of refined rapeseed oil—peroxide value (PV), anisidine value (*p*-AnV), acid value (AV), and spectrophotometric indices (K_232_, K_268_)—increased during storage. However, the addition of β-sitosteryl sinapate caused a decrease of the primary and secondary oxidation products in the supplemented oils in comparison with the control sample. Moreover, oils with steryl ester had higher AA than oil without the synthetic antioxidant. The accelerated storage negatively affected the antioxidant potential of refined and enriched oils causing the AA decrease by 25–54% and 7–15%, respectively. Studies have consistently demonstrated beneficial associations between the presence of β-sitosteryl sinapate in oil samples and the inhibition of their oxidative degradation under the accelerated conditions. Additionally, the possibility of using the synchronous fluorescence (SF) spectroscopy and excitation–emission matrix (EEM) fluorescence spectroscopy for identification and observing changes in main fluorescent components present in non-supplemented and supplemented rapeseed oils during the accelerated storage was attempted.

## 1. Introduction

Rapeseed oil, after palm oil and soybean oil, is one of the most popular vegetable oils in the world. Rapeseed oil is often recommended by dieticians as a valuable source of necessary polyunsaturated fatty acids and has a relatively good ratio of omega-6 to omega-3 fatty acids [1]. However, large amounts of unsaturated fatty acids (including oleic acid >61%, linoleic acid ~21%, and α-linolenic acid ~11%) present in rapeseed oil are a serious problem due to their easy oxidation processes that lead to the production of undesirable chemical compounds (aldehydes, ketones, and carboxylic acids), loss of shelf-life, safety, and nutritional value. It is well known that oxidation is the primary reaction responsible for the deterioration of oils during storage, which negatively affects their quality and sensory characteristics, mainly taste, color, and flavor.

On the other hand, crude rapeseed oil contains a high number of antioxidants, namely, tocopherols, carotenoid pigments, sterols, and phenolic compounds (mainly sinapic acid derivatives: sinapine-choline ester of sinapic acid and vinylsyringol (canolol)-decarboxylated product of sinapic acid) that enhance the oxidative stability of oil [2,3]. Unfortunately, natural antioxidants are partially removed from crude rapeseed oil during technological processes, especially refining. Therefore, the addition of antioxidant compounds to reduce the oxidation processes has been suggested. Antioxidants are required to delay uncontrolled reactions of oxidation, quench free radicals, and prolong usability and shelf life of vegetable oils.

The artificial phenolic antioxidants such as butylated hydroxyanisole (BHA), butylated hydroxytoluene (BHT), tertiary butylhydroquinone (TBHQ), and propyl gallate are widely used to prevent the oxidative rancidity of edible oils. As these synthetic antioxidants might cause a loss of nutrients, and even produce toxic and mutagenic effects, their safety is questioned [4].

Recently, great interest has been focused on using natural antioxidants having different polarity, such as tocopherols, ascorbyl palmitate, phenolic compounds, and other bioactive components present in plant extracts to improve oxidative stability and to prolong shelf life of rapeseed oil [5,6,7,8,9,10,11,12,13]. Some reports suggest that mixtures of polyphenols extracted from plant materials represent an important alternative to be used in the task of improving the oil stability. Nevertheless, oil industry waste (rapeseed meal, soapstock/acid oil, deodorized distillates, spent bleaching clay from oil refining) are sources of natural bioactive compounds (phenolics, lecithin, vitamin E) with antioxidant activity (AA) efficiently preventing the oxidative degradation processes of rapeseed oil [2,14,15,16].

However, the enrichment of oil systems with these natural compounds having polar nature and multi-ring structures can be limited due to their poor solubility in oils and low stability during processing. Therefore, to improve the lipophilicity of phenolic acids, esterification with aliphatic alcohols or phytosterols to amphiphilic molecules can significantly increase their solubility in non-aqueous media. Additionally, esterification of phytosterols with phenolic acids enhanced thermal stability of the synthesized antioxidants [17].

It is well known that phytosterols are predominantly present in oilseed plants, while rapeseed oil, after corn oil, is the second richest natural source of phytosterols (2.50–9.79 g/kg and 7.15–15.57 g/kg for rapeseed oil and corn oil, respectively) [18]. Sitosterol, brassicasterol, campesterol, and avenasterol are the most common phytosterols present in rapeseed and its products [19]. These bioactive compounds have attracted a great deal of attention due to their human health benefits, including cholesterol-lowering, antioxidant, anticancer, and anti-inflammatory functions. However, free phytosterols possess lower solubility in oil and higher melting points compared with phytosteryl esters [20,21,22]. For this reason, the esterification of phytosterols with phenolic acids is the most promising application of phytosteryl esters due to their easily incorporation into a variety of lipid matrices. Some authors reported the AA of phytosteryl phenolates in different model systems such as bulk oils, emulsions, and cooked pork [17,21,22,23]. Specifically, steryl ferulates are effective antioxidants that can protect vegetable oils from the oxidative degradation caused by thermal processes [17,23]. Our previous studies indicated that the total number and the configuration of hydroxyl and methoxy groups in aromatic ring mainly affected the antioxidant properties of hydroxycinnamate steryl esters [24]. Thus, β-sitosteryl sinapate (IC_50(DPPH)_ = 238.9 μmol/L and IC_50(ABTS)_ = 174.6 μmol/L) was found to be a more effective antioxidant than β-sitosteryl ferulate (IC_50(DPPH)_ = 290.0 μmol/L and IC_50(ABTS)_ = 206.0 μmol/L).

To the best of our knowledge, no systematic studies have been carried out on oxidative stability and the AA of refined rapeseed oils fortified with hydroxycinnamate steryl esters during storage. As a consequence, the purpose of the present work was to estimate the oxidative stability and antioxidant potential of refined rapeseed oil enriched with the novel antioxidant, β-sitosteryl sinapate, at a concentration of 200 ppm and isolated from light during accelerated shelf-life test. The oxidative parameters: peroxide value (PV), anisidine value (*p*-AnV), TOTOX index, acid value (AV), amounts of conjugated dienes (K_232_), and conjugated trienes (K_268_) were analyzed by the official procedures, whereas antioxidant potential was determined by three modified analytical methods: 2,2′-azino-bis(3-ethylbenzothiazoline-6-sulfonic acid) (ABTS), 2,2-diphenyl-1-picrylhydrazyl (DPPH) and Folin-Ciocalteu (FC) test. Additionally, synchronous fluorescence (SF) spectroscopy and excitation-emission matrix (EEM) fluorescence spectroscopy were used to observe the most characteristic qualitative changes in refined rapeseed oil without and with β-sitosteryl sinapate during the accelerated storage period.

Principal component analysis (PCA) was applied to determine the relationships between oils stored at recommended conditions and the variables (fluorescence features and chemical parameters) describing their properties.

## 2. Materials and Methods

### 2.1. Reagents and Materials

Sinapic acid, β-sitosteryl ≥70% (major impurities: campesterol and β-sitostanol), 2,2’-azino-bis(3-ethylbenzothiazoline-6-sulfonic acid) diammonium salt (ABTS), 2,2-diphenyl-1-picrylhydrazyl radical (DPPH, 95%), potassium persulfate, vanilin, acetic anhydride, 4-(dimethylamino)pyridine (DMAP), dicyclohexylcarbodiimide (DCC), 6-hydroxy-2,5,7,8-tetramethylchromane-2-carboxylic acid Trolox (TE), Folin-Ciocalteu reagent, potassium carbonate, and *p*-anisidine were obtained from Merck (Warszawa, Poland). Methanol, ethanol, ethyl acetate, dichloromethane, chloroform, *n*-hexane, anhydrous sodium carbonate, potassium iodine, starch, chloroform, acetic acid, and hydrochloric acid were provided by Chempur (Piekary Śląskie, Poland). TLC plates with fluorescent indicator UV_254_, trade name ALUGRAM^®^ SIL G/UV_254_ (Macherey-Nagel, Germany) and silica gel (pore size 60Å, Kieselgel, Macherey-Nagel, Germany) were purchased from Alchem (Toruń, Poland). Redistilled water was applied for preparation of all solutions.

### 2.2. Synthesis of β-Sitosteryl Sinapate

Amphiphilic antioxidant, β-sitosteryl sinapate was synthesized and purified by three steps synthesis proposed by Winkler-Moser et al. [25] with some modifications described in our previous report [24]. In the first step, sinapic acid was acetylated to 4-OH protected derivative. Then, esterification of the obtained 4-O-acetylsinapic acid with β-sitosteryl and deprotection of acetoxy group were performed, in the second and third steps, respectively. In brief:

1. Synthesis of 4-O-acetylsinapic acid

In a 50 mL round bottom flask equipped with a dropping funnel, thermometer, and magnetic stirrer, under nitrogen, sinapic acid (1.121 g, 5 mmol), and DMAP (13 mg, 0.1 mmol) were dissolved in pyridine (5 mL). Then, the solution was cooled to 0 °C, and acetic anhydride (0.562 g, 5.5 mmol) was added dropwise. The solution was stirred at 0 °C for 15 minutes and then at room temperature for 4 h. Water (5 mL) was added to the reaction mixture, and the solution was neutralized with 2 M HCl. The precipitate was separated and washed with distilled water. The crude product was suspended in methanol (10 mL), heated to reflux for 15 min, and then left to crystallize for 1 h at room temperature. The product was isolated and dried to give 4-O-acetylsinapic acid (1.198 g, yield 90%).

^1^H NMR (700 MHz, CDCl_3_) δ ppm: 2.35 (s, 3H), 3.86 (s, 6H), 6.40 (d, *J* = 15.9 Hz, 1H), 6.80 (s, 2H), 7.72 (d, *J* = 15.9 Hz, 1H). ^13^C NMR (100 MHz, CDCl_3_) δ ppm: 20.39, 56.19 (2 × OCH_3_), 104.94 (2 × CH), 117.54, 130.79, 132.28, 146.72, 152.47 (2 × C-OMe), 168.44, 172.02.

2. Synthesis of β-sitosteryl (4-O-acetyl)sinapate

4-O-Acetylsinapic acid (1.065 g, 4 mmol) and β-sitosteryl (1.244 g, 3 mmol) were dissolved in dichloromethane (25 mL) at 0 °C under nitrogen. Then, DMAP (0.122 g, 1 mmol) was added to the solution, followed by DCC (0.825 g, 8.1 mmol). The reaction was carried out at 0 °C for 30 min and room temperature for 1 h. *n*-Hexane (70 mL) was added, and precipitated 1,3-dicyclohexylurea was filtered off. The filtrate was evaporated on a rotary evaporator (Laborota 4003, Heidolph Instruments, Schwabach, Germany), and β-sitosteryl (4-O-acetyl)sinapate (1.671 g; 84%) was purified by flash column chromatography (Chemland, Stargard Szczeciński, Poland) (*n*-hexane/dichloromethane/ethyl acetate; 3:1:1).

^1^H NMR (700 MHz, CDCl_3_) δ ppm: 0.69 (s, 3H), 0.82 (d, *J* = 6.9 Hz, 3H), 0.84 (d, *J* = 6.7 Hz, 3H), 0.85 (t, *J* = 7.4 Hz, 3H), 0.93 (d, *J* = 6.5 Hz, 3H), 1.05 (s, 3H), 1.08–1.38 (m, 14H), 1.43–1.70 (m, 9H), 1.82–2.04 (m, 5H), 2.34 (s, 3H), 2.41 (m, 1H), 3.85 (s, 6H), 4.72–4.79 (m, 1H), 5.40–5.43 (m, 1H), 6.37 (d, *J* = 15.9 Hz, 1H), 6.77 (s, 2H), 7.60 (d, *J* = 15.9 Hz, 1H). ^13^C NMR (100 MHz, CDCl_3_) δ ppm: 11.87, 11.98, 18.79, 19.05, 19.34, 19.81, 20.42, 21.05, 23.09, 24.30, 26.12, 27.91, 28.25, 29.18, 31.90, 31.93, 33.96, 36.16, 36.64, 37.03, 38.24, 39.74, 42.33, 45.86, 50.07, 56.06, 56.19 (2 × OCH_3_), 56.71, 74.22, 104.65 (2 × CH), 119.02, 122.79, 130.36, 132.85, 139.62, 144.10, 152.42 (2 × C-OMe), 166.12, 168.46.

3. Synthesis of β-sitosteryl sinapate

For the synthesis of β-sitosteryl sinapate, K_2_CO_3_ (0.069 g, 0.5 mmol) was added to the solution of β-sitosteryl (4-O-acetyl)sinapate (1.657 g, 2.5 mmol) in chloroform:methanol (2:1, 100 mL) under nitrogen, and the solution was refluxed for 8 h. Next, the solution was cooled to room temperature and saturated aqueous NH_4_Cl (5 mL) was added. Then, dichloromethane (25 mL) was added and the organic layer was washed with water (2 × 20 mL) and saturated solution of NaCl (20 mL). Organic mixture was dried with MgSO_4_, filtered, and evaporated. β-Sitosteryl sinapate was purified by flash column chromatography (*n*-hexane/dichloromethane/ethyl acetate; 6:3:1) to yield 1.350 g—87%.

^1^H NMR (700 MHz, CDCl_3_) δ ppm: 0.69 (s, 3H), 0.82 (d, *J* = 6.7 Hz, 3H), 0.84 (d, *J* = 6.9 Hz, 3H), 0.85 (t, *J* = 7.4 Hz, 3H), 0.93 (d, *J* = 6.5 Hz, 3H), 1.05 (s, 3H), 1.08–1.37 (m, 14H), 1.43–1.70 (m, 9H), 1.81–2.04 (m, 5H), 2.40 (m, 1H), 3.92 (s, 6H), 4.72–4.78 (m, 1H), 5.41 (d, *J* = 5.2 Hz, 1H), 5.74 (s, 1H), 6.29 (d, *J* = 15.9 Hz, 1H), 6.77 (s, 2H), 7.58 (d, *J* = 15.7 Hz, 1H). ^13^C NMR (100 MHz, CDCl_3_) δ ppm: 11.85, 11.97, 18.78, 19.05, 19.31, 19.79, 21.04, 23.10, 24.29, 26.17, 27.94, 28.22, 29.21, 31.90, 31.92, 33.97, 36.15, 36.64, 37.05, 38.29, 39.75, 42.33, 45.88, 50.08, 56.08, 56.32 (2 × OCH_3_), 56.71, 73.95, 105.08 (2 × CH), 116.51, 122.69, 126.06, 137.10, 139.69, 144.64, 147.23 (2 × C-OMe), 166.47.

**Figure 1:**
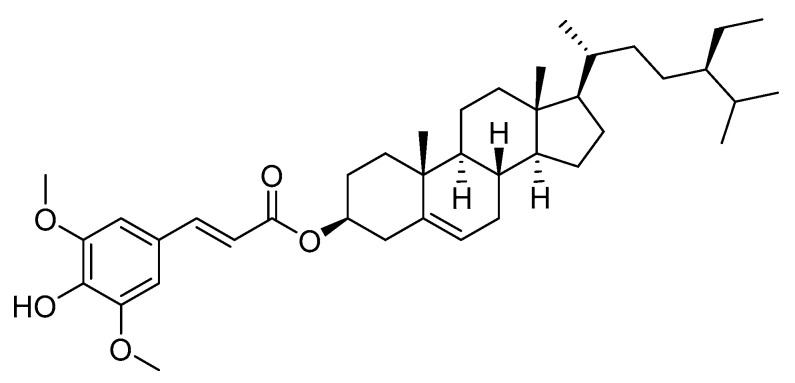
Structure of β-sitosteryl sinapate.

Structure of β-sitosteryl sinapate was presented in Figure 1.

### 2.3. Accelerated Storage of Oil Samples

The freshly refined rapeseed oil in the original polypropylene containers was kindly donated by a local vegetable oil factory. 

The synthesized and purified β-sitosteryl sinapate (0.1 g) was added to rapeseed oil (500 g) and stirred for 5 min to obtain a final concentration of 200 ppm. Then, 100 mL of rapeseed oil fortified with β-sitosteryl sinapate was transferred to four transparent glass bottles covered with aluminum foil (6.2 cm × 2.9 cm × 12.7 cm) to prevent passage of light through these bottles. For comparison, four rapeseed oil samples without synthetic antioxidant, after 5 min of stirring at room temperature were introduced to the same transparent glass bottles covered with aluminum foil. 

**Figure 2:**
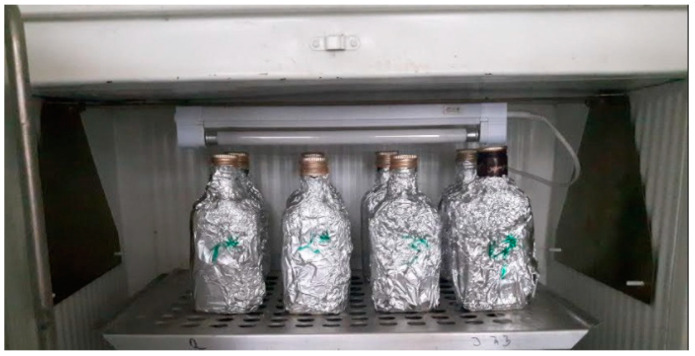
Position of oil samples in incubator.

The accelerated shelf-life experiment was conducted according to the procedure described in our previous work [26]. Briefly, eight oil samples were stored in an incubator (Elkon CWE-2a, Łódź, Poland) at a distance of 300 mm from the fluorescent lamp (T5 8W F8W/33 GE, power of luminous flux = 385 lm). Each row of bottles with oils was positioned at a distance of 25 mm from the fluorescent tube (length = 380 nm) as shown at Figure 2 and incubated for 4 weeks at 40 °C. Samples were withdrawn at intervals of one week for analysis. These experimental conditions were used to simulate (mimic) a real storage of refined rapeseed oils in a retail place for recommended 12-month shelf life (each week of oils’ storage under proposed accelerated conditions can be considered as storage period of 3 months at 10–15 °C). The lighting and temperature (40 °C) of all samples throughout the experiment were strictly controlled.

### 2.4. Chemical Analysis of Oil Samples

The primary and secondary oxidation products expressed as peroxide value (PV) and *p*-anisidine value (*p*-AnV) were analyzed by the official methods: International ISO 3960:2017 [27] and ISO 6885:2016 [28], respectively. The oxidation state of oil given by the TOTOX index was calculated according to the formula: (TOTOX = 2PV + *p*-AnV). Extinction coefficients (K_232_ and K_268_) were measured by UV-Vis spectrophotometer (Hitachi U-2900, Tokyo, Japan) as the absorbance of 1% solution of each oil in *n*-hexane at 232 and 268 nm, respectively, in 1-cm cell path length. Both extinction coefficients were determined according to ISO 3656:2011 method [29]. The degree of oil hydrolysis was assessed by measuring the acid value (AV) and free fatty acids (FFA) using ISO 660:2020 method [30].

### 2.5. Antioxidant Activity of Oil Samples

Methanolic extracts of rapeseed oils without and with β-sitosteryl sinapate were prepared according to the previous procedure [26]. In brief, 2.00 g of each oil was extracted with 5 mL of methanol and extraction were carried out in a shaker SHKA 2508-1CE (Labo Plus, Warszawa, Poland) at ambient temperature in the dark for 30 min. In order to separation of extracts from oils, the samples were stored at –20°C for 30 min. Extractions were repeated three times, and the combined extracts were transferred quantitatively into glass bottles.

The AA of non-supplemented and supplemented rapeseed oils were analyzed by three modified spectrophotometric methods: ABTS, DPPH, and FC described previously [31]. The UV-Vis spectra were recorded using a Hitachi U-2900 spectrophotometer (Hitachi, Tokyo, Japan) in a 1 cm quartz cell. The AA of oil samples was expressed as micromoles of Trolox equivalents per 100 g of sample. 

### 2.6. Fluorescence Studies of Oil Samples

Fluorescence spectra were obtained by Gilden pλotonics fluoroSENS instrument (Gilden Photonics Ltd., Glasgow, UK) equipped with a xenon lamp and connected to a personal computer. Instrumental parameters were set in our previous work [32]. Each oil sample diluted in *n*-hexane (1%) was set in a 10 mm quartz cuvette. The fluorescence excitation-emission matrix (EEM) was obtained for each sample with both the excitation and the emission bandwidths set at 10 nm for measurement ranges between 250 and 450 nm, and 250 and 700 nm, respectively. Rayleigh signals were removed in all EEM by inserting the zero in regions, where λ_em_ ≤ λ_exc_ and λ_em_ ≥ 2 × λ_exc_. Synchronous fluorescence (SF) spectra were acquired by simultaneous scanning of the excitation and the emission monochromators, with a constant distance, Δλ of 30 nm. All analyses were carried out in triplicate, and the results reported as mean values. Fluorescence intensities were plotted as a function of the excitation wavelength.

### 2.7. Statistical Analysis

The oxidation parameters: PV, *p*-AnV, K_232_, K_268_, and AV for each oil sample were analyzed three times (three portions of oil was taken from the same bottle) within 1 day after each week of storage. However, the AA of rapeseed oils before and after accelerated storage were determined (five portions of each methanolic extract analyzed within 1 day) by the modified ABTS, DPPH, and FC assays. All calculated data were reported as mean (c) ± standard deviation (SD). The influence of storage conditions on the chemical properties of the studied oils was assessed by means of one-way analysis of variance (ANOVA), followed by a Duncan’s post hoc multi-comparison test at the significance level 0.05. The Pearson correlation analysis was used to determine correlations among variables (PV, *p*-AnV, TOTOX, K_232_, K_268_, AV, FFA, ABTS, DPPH, FC, and fluorescence spectral features).

PCA was used to acquire a better knowledge on evolution of fluorescence spectra and chemical parameters of studied oils over time as oxidation progresses. The spectral features in the range of 300–550 nm, oxidation parameters (PV, *p*-AnV, TOTOX, K_232_, K_268_, AV, FFA), and the AA results (ABTS, DPPH, FC) of investigated oils were used as active variables in the derivation of the principal components, and the various oil samples (refined rapeseed oils without and with steryl ester stored during 4 weeks) were represented on bi-plot.

Statistical analysis of data was performed using the Statistica 8.0 software (StatSoft, Tulsa, OK, USA).

## 3. Results and Discussion

### 3.1. Changes in Chemical Parameters of Rapeseed Oils Without and With Steryl Ester during Accelerated Storage

The oxidative status of refined rapeseed oils without and with β-sitosteryl sinapate was evaluated during accelerated shelf-life test by characteristic values such as PV, *p*-AnV, TOTOX, K_232_, K_268_, AV and FFA (Table 1).

Initial PV values were 0.10 and 2.24 meq O_2_/kg for control oil without β-sitosteryl sinapate and oil enriched with 200 ppm of this synthetic antioxidant, respectively. Probably, the presence of a new antioxidant, β-sitosteryl sinapate, in refined rapeseed oil intensively stirred without nitrogen protection during the oil fortification caused the significant increase in its initial PV.

This experiment demonstrated a linear increase (correlation coefficients, r ranged between 0.9804 and 0.9900) in the PV results of non-supplemented and supplemented rapeseed oils during accelerated test (4 weeks, T = 40 °C, fluorescent lamp). 

It is noteworthy that the amount of primary oxidation products in refined rapeseed oil after the second week of storage was above the maximum value (PV < 5 meq O_2_/kg) permitted for vegetable oils according to the ISO 3960 [27]. However, the incorporation oil with a new steryl ester significantly (Duncan test) limited the generation of hydroperoxides (PV = 6.00 meq O_2_/kg after 4 weeks of storage). Hydroperoxide contents in the enriched rapeseed oils were approximately 2 times lower in comparison with the control oil samples after storage the same period (Table 1).

Some natural extracts of *Bifurcaria bifurcata*, *Sorbus aucuparia* (L.) and *Malus baccata* (L.), *Teucrium polium* essentials oil, tomato waste as well as synthetic compounds: caffeic acid amide, octyl sinapate, and commercially available BHA may result in a protective effect against oxidative processes of rapeseed oils during storage [8,9,26,33,34,35]. The formation of hydroperoxides in rapeseed oil fortified with *Bifurcaria bifurcata* extract after 16 days of accelerated storage was about 4 times lower than in control sample. Inhibitory effect of *Bifurcaria bifurcata* extract against primary oxidation increased with the increase in the extract’s concentration (from 200 to 1000 ppm), whereas 200 ppm of BHA and 600 ppm of *Bifurcaria bifurcata* extract had similar protective effect against hydroperoxides generation [8]. In contrary, minor differences between PV values for rapeseed oils with octyl sinapate (PV = 1.31–93.13 meq O_2_/kg) and refined oil (PV = 0.02–99.87 meq O_2_/kg) were found in our previous studies [26].

However, hydroperoxides as primary products of lipid oxidation are labile and decompose rapidly to different secondary products. As seen, the *p*-AnV results of fresh oils without and with a new antioxidant ranged between 0.71 and 0.77, and they significantly differ from each other (Table 1, Duncan test). The increases in the *p*-AnV with increasing storage time indicates the generation of secondary oxidation products in rapeseed oil samples. Although, the addition of β-sitosteryl sinapate retarded the transformation of odorless and colorless primary oxidation products into secondary oxidation products such as aldehydes, ketones, alcohols, acids, hydrocarbons, etc. The highest increase of *p*-AnV (0.77–2.12) for non-supplemented oil was observed during the first week of storage. However, steryl ester stabilized secondary oxidation products, thus the *p*-AnV for fortified oil (4.54) was approximately 2 times lower than this obtained for control oil (*p*-AnV = 10.42) after the accelerated storage test. The chemical structure of β-sitosteryl sinapate, especially the presence of phenolic hydroxyl and methoxy groups inhibited the rate of propagation and other oxidative reactions (Figure 1). It can be noted that *p*-AnV results for refined oils with β-sitosteryl sinapate were within the desirable level for refined vegetable oils (*p*-AnV < 8) [28], whereas total content of secondary oxidation products in oils without antioxidant were significantly higher after the third week (*p*-AnV = 8.44–10.42).

Similar effects of synthetic and natural antioxidants on the formation of the secondary oxidation products in rapeseed oils during storage were reported by other authors [8,26,32]. After 4 weeks of storage at 40 °C under light, the *p*-AnV of rapeseed oil with octyl sinapate decreased by approximately 34% compared with the oil without synthetic antioxidant [26]. However, rapeseed oils enriched with phenolic antioxidants from *Bifurcaria bifurcata* extract exhibited *p*-AnV results lower by 55–68% than control sample [8].

Total oxidation values (TOTOX) combining the amounts of primary with secondary oxidation products were applied to estimate oxidative deterioration of rapeseed oils without and with β-sitosteryl sinapate under the accelerated conditions. Unfortunately, this factor was within desirable level (<10) only for the first 7 days of storage (TOTOX = 7.99 and 9.40 for non-supplemented and supplemented oils, respectively). It is evident that the accelerated conditions (T = 40 °C and fluorescence light = 385 lm) significantly affected the overall oxidation of rapeseed oils (Table 1). However, the enrichment of oil with β-sitosteryl sinapate gave rise to lower increment of TOTOX values (5.19–16.54 for oils with antioxidant and 4.79–35.24 for oils without steryl ester). Differences in TOTOX results between non-supplemented and supplemented oils were noticeable from the second week due to the significant protective effect of the new antioxidant.

Furthermore, K_232_ and K_268_ are suitable parameters for evaluation of oxidative deterioration of oil. The addition of β-sitosteryl sinapate caused a not statistically significant increase in K_232_ of the studied oils under accelerated storage (Duncan test, Table 1). This suggests that the proposed phenolic antioxidant has been shown to be effective against rapeseed oil oxidation measured as K_232_ and K_268_. In contrast, higher amounts of conjugated dienes (K_232_ = 2.775–2.882) and conjugated trienes (K_268_ = 0.522–0.704) in refined rapeseed oils without antioxidant differed significantly after each week of the accelerated storage.

For comparison, rapeseed oils enriched with celery and *Bifurcaria bifurcata* extracts as well as synthetic antioxidants: TBHQ and BHT had lower levels of conjugated dienes and conjugated trienes than control samples [8,10]. At the end of storage experiments, the number of conjugated dienes in rapeseed oil fortified with 1000 ppm of *Bifurcaria bifurcata* extract was reduced by about 73% [8].

Additionally, the Duncan test indicated that AV results and FFA content in rapeseed oils without and with β-sitosteryl sinapate stored under accelerated conditions were significantly different from each other (Table 1). However, all studied oils had AV below the desirable level (AV < 0.300 mg NaOH/g) for refined oils [30]. Although, an increase in the AV (0.0487–0.1257 and 0.0480–0.0864 mg NaOH/g for refined and fortified rapeseed oils, respectively) and FFA (0.0344–0.0888 and 0.0339–0.0610% for refined and fortified rapeseed oils) with increasing time of their storage was observed. This fact can be explained in that free fatty acids were formed due to hydrolysis or lipolysis as a result of triacylglycerols break down during storage of the studied oils. It is noteworthy that the addition of a synthetic steryl ester delayed the undesirable reactions (Table 1).

Additionally, the formation of FFA was slower in rapeseed oils with celery extract (AV = 0.1–0.3 mg/g) and TBHQ (AV = 0.1–0.6 mg/g) than in control oil sample (AV = 0.1–1.6 mg/g) stored under thermal conditions for 24 h [10].

### 3.2. Changes in Antioxidant Activity of Rapeseed Oils Without and With Steryl Ester during Accelerated Storage

The AA of oils are directly related to presence of compounds with antioxidant properties that had reducing power and they are capable to either delay or inhibit the oxidation processes. The natural and added antioxidants prevent oxidative rancidity of vegetable oils and increase their nutrient value. For this reason, the AA of refined rapeseed oils before and after addition of β-sitosteryl sinapate during 4 weeks of storage at 40 °C under fluorescent lamp were determined by three modified spectrophotometric methods: ABTS, DPPH and FC and the obtained results were listed in Table 2.

As seen, the AA of rapeseed oils analyzed during 4 weeks of storage at accelerated conditions by three proposed analytical tests differ significantly (Table 2). Probably the different mechanisms of the used analytical methods affect these discrepancies between the AA results. The refined rapeseed oil enriched with a new antioxidant had significantly higher ABTS (2095.8–2504.6 µmol TE/100 g), DPPH (748.6–806.2 µmol TE/100 g), and FC (117.2–132.3 µmol TE/100 g) results than oils without the new antioxidant (ABTS = 1346.8–2384.8 µmol TE/100 g, DPPH = 452.6–606.0 µmol TE/100 g, and FC = 59.1–129.4 µmol TE/100 g). Regardless of the analytical assay performed, the significant decrease in AA of refined rapeseed oils without β-sitosteryl sinapate was observed throughout the storage time (Duncan test, Table 2). Nevertheless, fortification of oils with β-sitosteryl sinapate caused a not statistically significant decrease in their AA between 21 and 28 days of storage. This suggests that degradation of antioxidants in oils after incorporation of the synthesized steryl ester was minimal, specifically in the last stage of the accelerated storage, while unstable natural antioxidant components in control refined rapeseed oil were degraded. The accelerated storage resulted in significantly lower losses of ABTS (15%), DPPH (7%), and FC (11%) for oil with the synthesized antioxidant than those for the control sample (the reduction of ABTS = 44%, DPPH = 25%, and FC = 54%). Therefore, freshly (0-week storage) refined rapeseed oil without a synthetic antioxidant revealed only somewhat higher ABTS and FC values, but significantly lower DPPH result in comparison with the AA of oil fortified with steryl ester after 28 days of the accelerated storage (Duncan test, Table 2). Nevertheless, antioxidative properties of oils before and after supplementation of steryl ester decreased significantly and linearly (r = 0.9404–0.9958 and 0.8864–0.9504, respectively, *p* = 0.00033–0.045) with storage time under accelerated conditions. Our previous report [24] confirmed that the β-sitosteryl sinapate has significant antioxidant potential (IC_50(ABTS)_ = 174.6 µmol/L and IC_50(DPPH)_ = 238.9 µmol/L); hence, its addition to rapeseed oil creates effective defense system against free radical attack. The reduction of antioxidant potential of rapeseed oil may be delayed by the addition of this amphiphilic steryl ester having one hydroxyl and two methoxy groups (Figure 1), which possess the ability to breaking the free radical chain reaction by donating H-atom (s).

For comparison, during the same storage period at the same conditions, higher decrease in ABTS (23%), DPPH (27%), and FC (20%) for rapeseed oil enriched with 0.9% of octyl sinapate was found [26]. Moreover, canola oils incorporated with different concentrations (200–1200 ppm) of *Teucrium polium* essential oil and BHA (200 ppm) and incubated for 60 days at room temperature had a significantly higher total content of phenolics (TPC = 110–125 mg gallic acid (GA)/g) than the control sample without antioxidants (95–105 mg GA/g), although the reduction of TPC in each oil sample with or without antioxidants with enhancing time was observed [35]. In addition, rapeseed oils with 0.25–1.5% of lovage stems, lovage leaves, and horseradish leaves extracts, as well as 0.01% of BHT had a slower decrease in DPPH values (approximately 1.6–2 times) than for the control rapeseed oil (about 3 times) during storage in the dark at 60 °C was found by Tomsone and Krūma [36].

### 3.3. Fluorescence Characteristics of Rapeseed Oils Without and With Steryl Ester during Accelerated Storage

**Figure 3:**
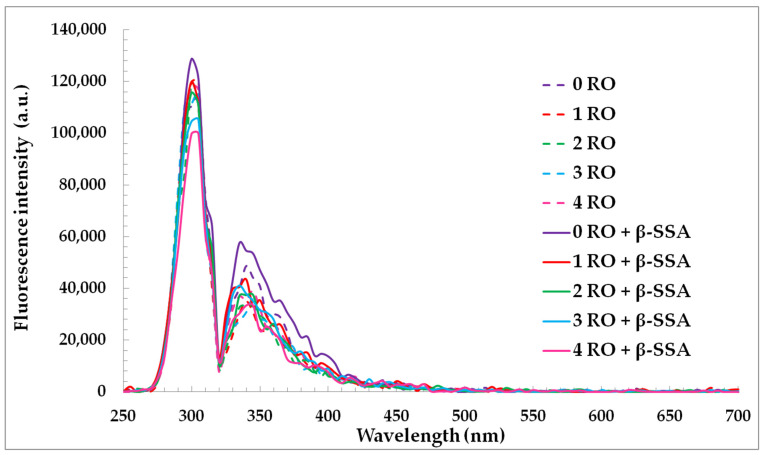
Synchronous fluorescence spectra of refined rapeseed oils without and with β-sitosteryl sinapate diluted in *n*-hexane (c = 1%) and recorded at Δλ = 30 nm after storage at accelerated conditions.

The synchronous fluorescence (SF) spectra of rapeseed oils before and after fortification of β-sitosteryl sinapate diluted in *n*-hexane were used for characterization of changes in oils’ components during accelerated storage (Figure 3).

**Figure 4:**
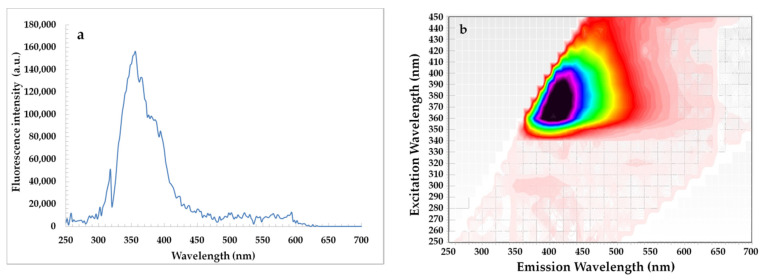
Synchronous fluorescence spectrum recorded at Δλ = 30 nm (**a**) and excitation-emission matrix (**b**) of β-sitosteryl sinapate diluted in *n*-hexane (c = 0.1%).

Changes in SF spectra under accelerated conditions have been related to degradation of fluorescent components naturally present in rapeseed oil, mainly tocopherols, phenolic compounds and chlorophyll. As seen, each SF spectrum of oil had a relatively intense bands in the emission range of 300–360 nm ascribed to tocopherols and phenolic compounds [37]. The fluorescence maxima of the short wavelength emission at 300 nm and 330–360 nm for the studied oils gradually decreased after each week of storage under accelerated conditions (T = 40 °C, fluorescent lamp) (Figure 3). This can be explained by the fact that accelerated conditions caused the changes in amounts of tocopherols and phenolic compounds in rapeseed oils without and with steryl ester. Therefore, each week of storage led to a decrease in the ABTS, DPPH, and FC results of the investigated oils (Table 2). Although, the SF spectra for rapeseed oils after the addition of steryl ester revealed a stronger band in the region between 330 and 360 nm characteristic for both phenolic compounds and β-sitosteryl sinapate absorption (Figure 4a).

A slower decreasing of fluorophores concentrations (natural phenolics in presence of steryl ester) under the accelerated test was then, indeed, due to a lower decrease in the AA of enriched oils (Table 2). Interestingly, the peak at 300 nm of rapeseed oils incorporated with steryl ester had lower intensity values due to faster consumption of the natural tocopherols in the presence of new synthetic antioxidant under accelerated conditions (Figure 3). The obtained results suggest that SF spectra can be applied for non-destructive, quick, and simple monitoring of changes in fluorescence antioxidants and their decomposition.

The SF spectroscopy was also used by other authors to characterize and evaluate the quality of various vegetable oils based on their degree of oxidation [32,37,38,39,40,41,42,43]. The SF spectra of vegetable oils exhibited that the fluorescence bands attributed to tocopherols, chlorophylls, and pheophytins in the ranges of 300–370 nm and 550–700 nm, respectively, decreased during oxidation stages under various conditions. However, fluorescence signals between 350 and 550 nm associated with hydrolysis and oxidative products significantly increased in degraded oils. A significant reduction of fluorescence intensity at 330 nm (degradation of tocopherols) and a slight increase of fluorescence at 360 and 420 nm (formation of polar compounds) in the spectra of rapeseed oil heated at 171 °C and 189 °C for 133 and 283 min were observed by Mas et al. [40]. Nevertheless, the band at 300 nm for rapeseed oils incorporated with 200 ppm of synthetic antioxidants such as octyl sinapate and BHA was much stronger, but showed an overall downward trend during storage under different conditions [32]. This suggests that the added antioxidant inhibited the degradation of tocopherols and regenerated these natural components by the reduction of tocopheroxyl radicals. Although, an exogenous antioxidant added in low concentration (200 ppm) did not protect the rapeseed oil from generation of oxidation products during storage. Therefore, fluorescence spectra of rapeseed oils without and with 200 ppm of octyl sinapate revealed low intense peaks between 400 and 500 nm characteristic for oxidation products [32].

The not statistically significant fluorescence intensities changes in the range of 400–520 nm can be explained by the generation of low amounts of primary (PV = 0.10–12.41 meq O_2_/kg, K_232_ = 2.415–2.882, K_268_ = 0.522–0.704) and secondary (*p*-AnV = 0.71–10.42) oxidation products in rapeseed oils stored in glass bottles covered with aluminum foil under accelerated conditions (Figure 3, Table 1).

**Figure 5:**
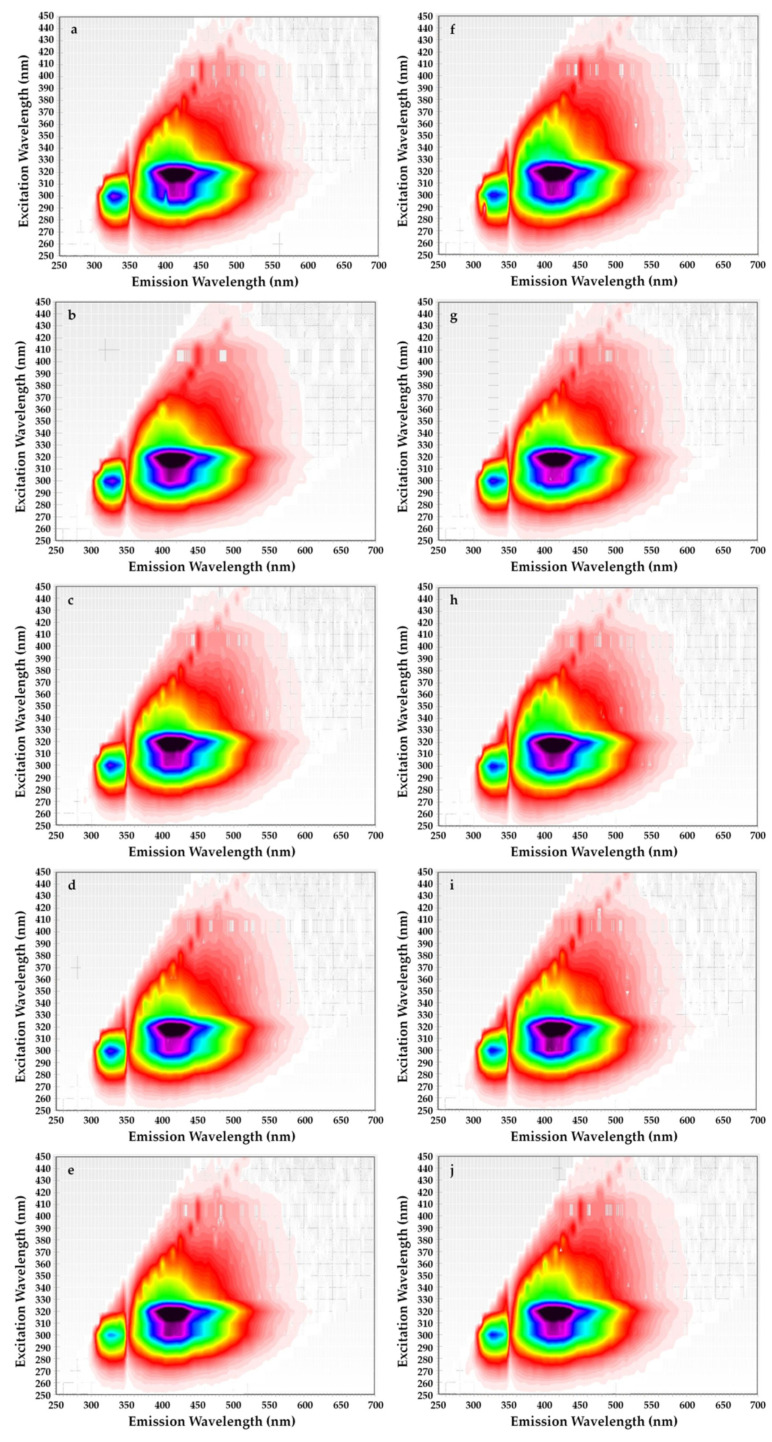
Excitation–emission matrices of refined rapeseed oils without β-sitosteryl sinapate (**a**–**e**) and refined rapeseed oils with β-sitosteryl sinapate (**f**–**j**) after 0, 1, 2, 3 and 4 weeks of storage at accelerated conditions.

In addition, the two-dimensional excitation–emission matrices (EEM) fluorescence spectroscopy of rapeseed oils before and after supplementation with steryl ester contained the changes in excitation and emission profiles of the fluorescent components in each oil sample for a period of 4 weeks (Figure 5).

The not statistically significant differences in the shape and intensities of the fluorescence bands on EEM for rapeseed oil without exogenous antioxidant and after spiked with β-sitosteryl sinapate were found. All oil samples revealed a strong characteristic band with excitation at 270–320 nm and emission at 330–350 nm attributed to tocopherols (Figure 5). However, tocopherols subsequently decomposed in rapeseed oil after each week of storage at accelerated conditions (T = 40 °C, fluorescent lamp). For this reason, this characteristic band for oils without and with 200 ppm of steryl ester exhibited the lowest fluorescence intensity after 28 days of storage (Figure 5e,j). Unexpectedly, the addition of a new antioxidant led to a decrease in the fluorescent band corresponding to tocopherols (Figure 5f–j). This fact can be explained as a synergistic interaction between naturally present tocopherols and phenolics as well as added steryl sinapate.

The decrease in intensity of the peak attributed to tocopherols (λ_exc_/λ_em_ = 300/330 nm) was also depicted in the EEM spectra of refined rapeseed oils incorporated with 0.02–0.5% of octyl sinapate and 0.02% of BHA [32]. Moreover, the most intense fluorescent bands in the EEM spectrum of freshly pressed rapeseed oil corresponding respectively to tocopherols (λ_exc_/λ_em_ = 300/331 nm) and pheophytins (λ_exc_/λ_em_ = 400/680 nm) decreased considerably in the EEM spectra of samples stored for 6 months in darkness as well as exposed to light in green and colorless bottles [39].

On the other hand, the EEM plots indicated that all studied oils had similar content of oxidation products due to the shape having a maximum intensity at λ_exc_/λ_em_ = 320/400 nm did not differ significantly. Although amounts of primary and secondary oxidation products in rapeseed oils without and with steryl ester determined by official methods significantly increased with the extension of storage time (Table 1). Obviously, levels of primary and secondary oxidation products were lower in oils fortified with steryl ester than in oils without synthetic antioxidant (Table 1), but the EEM spectra (Figure 5f–j) did not demonstrate the inhibitory effect of steryl ester added to rapeseed oil at a concentration of 200 ppm against oxidation processes. Furthermore, the EEM spectrum of β-sitosteryl sinapate diluted in *n*-hexane (c = 0.1%) had a strong peak at λ_exc_/λ_em_ = 380/410 nm (Figure 4b), whereas this band was not observed in EEM spectra of rapeseed oils enriched with 200 ppm of new antioxidant (Figure 5f–j). This suggests that the intensities of the peaks for endogenous antioxidants present in rapeseed oil were stronger than the intensity of peak for exogenous antioxidant added at low concentration of 200 ppm, hence further studies are needed to identify the respective fluorophores.

In contrast, the peak with the fluorescence intensity in the EEM spectra at excitation/emission of 320/400 nm for refined rapeseed oils without and with 0.02% of octyl sinapate increased during storage at refrigerated temperature and exposure to UV light, whereas this band reduced in oil samples with 0.5% of OSA and 0.02% [32].

Moreover, a broad fluorescence band in the intermediate region (λexc/λem = 320/400 nm) associated with polar compounds and degradation products formed in the oxidation reactions was observed in EEM spectra of cold-pressed rapeseed oils exposed to light for 6 months in green and colorless bottles [39].

### 3.4. Principal Component Analysis

**Figure 6:**
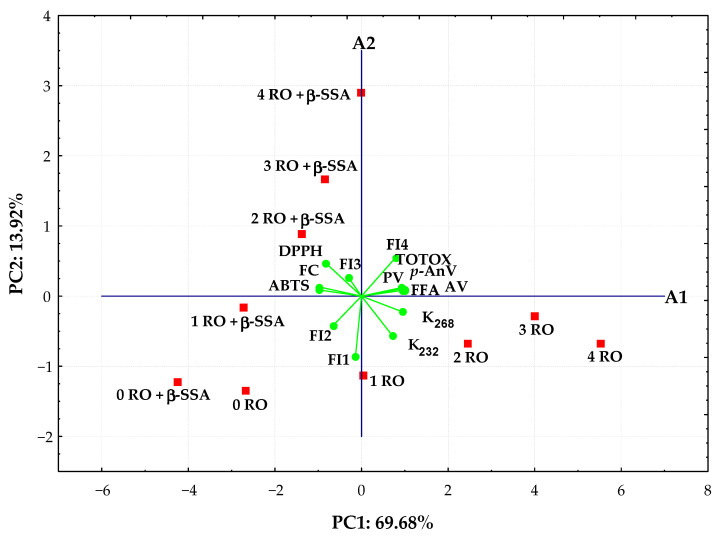
Bi-plot of scores and loadings of data obtained from fluorescence spectra, oxidative, and antioxidant parameters of refined rapeseed oils without and with β-sitosteryl sinapate stored during during 4 weeks.

PCA was applied to analyze values of synchronous fluorescence intensity measured in the selected range 300–550 nm at Δλ = 30 nm and the analytical parameters of 10 rapeseed oils to better understand the evolution of fluorescence spectra and changes in chemical properties of oils over time as oxidation progresses. The scores for the studied rapeseed oil samples and the distribution of the most significant variables in the first two principal components that explain 83.60% of the data matrix variance was depicted in the bi-plot (Figure 6).

As seen, the distribution of rapeseed oils in the bi-plot depended on the presence of new antioxidant in samples and storage time. A more oxidized non-supplemented rapeseed oils stored during 4 weeks with high oxidation parameters and fluorescence bands between 400 and 550 nm, but low AA results were located to the right in the score plot and had positive PC1 and negative PC2 values. The other group, situated in the left side with negative values for PC1, was represented by fresh oils and oils incorporating steryl ester that exhibited high antioxidant properties and intense fluorescence maxima of the short wavelength emission between 300 and 360 nm. The fresh oils (0 RO and 0 RO + β-SSA) and fortified oil after 1 week of storage (1 RO + β-SSA) characterized by the lowest oxidation parameters and FI4 fluorescent component related to the oxidation products emission created evidently distinct cluster (Figure 6). Moreover, the stored oils containing synthetic antioxidant with high ABTS, DPPH, and FC results as well as FI3 fluorescent component related to both phenolic compounds and β-sitosteryl sinapate absorption were located upper the A1 axis.

It can be noted that the studied oil samples spread along the PC1 axis, from negative to positive values, according to the storage time.

The PC1 positively correlated (correlation loadings > 0.7) with all oxidative parameters and FI4 fluorescent component (oxidation products) variables, whereas PC2 was highly negatively contributed by FI1 fluorescent component (r = −0.858) related to tocopherols emission. Evidently, PC1 is generally more correlated with the variables than PC2.

Moreover, the FI4 fluorescent component identified as oxidation products in the studied oils was significantly positively correlated to their oxidative parameters (r = 0.7445–0.7942, *p* < 0.02), while there were negative correlations between FI4 and two fluorescent components (FI1 and FI2) related to tocopherols and phenolic compounds (r varied from −0.6338 to −0.7907, *p* < 0.05) as well as ABTS (r = −0.7286, *p* = 0.0169) and FC (r = −0.6746, *p* = 0.0324) results. A significant positive correlation (r = 0.6360–0.9888, *p* < 0.05) for all determined oxidation parameters was calculated. Although, these oxidative values correlated significantly negatively with AA results of oil samples (r ranged between −0.6443 and −0.9836, *p* < 0.05). However, high positive correlation coefficients (r = 0.8179–0.9620, *p* < 0.005) were observed between antioxidant potential of oils analyzed by the modified ABTS, DPPH, and FC methods.

The SF spectra combined with PCA could be used not only to differentiate various oils without and with β-sitosteryl sinapate, but also to monitor their oxidation evolution during storage under the accelerated conditions.

## 4. Conclusions

An amphiphilic antioxidant, β-sitosteryl sinapate was synthesized using the modified three step chemical synthesis strategy. The supplementation of rapeseed oil with a new antioxidant extended its shelf life estimated during accelerated test. Rapeseed oils containing 200 ppm of β-sitosteryl sinapate were identified as more oxidative stable than control samples without amphiphilic antioxidant, suggesting a high antioxidant potential of synthesized compound against primary and secondary oxidation products after 4 weeks of the accelerated storage. Significantly higher AA values for rapeseed oils with steryl ester determined by ABTS, DPPH, and FC methods decreased slowly under accelerated conditions.

Effect of added antioxidant on the changes in fluorescent components naturally present in rapeseed oil and its oxidative degradation during the 4-week studies at 40 °C under light was reflected in the SF and EEM spectral characteristics. Fluorescence spectroscopy is a rapid and non-destructive technique for the monitoring of oil degradation, whereas the novel amphiphilic antioxidant offers an intriguing solution for hydrophilic phenolic antioxidants and can have great potential application in fat products.

However, further studies are warranted to fully investigate the cytotoxicity effect of a new antioxidant, β-sitosteryl sinapate against both normal and cancer cells. Especially important will be studies on the in vivo antiproliferative activities of new compounds on animal models. This approach will indicate the effective concentration of novel compound for inhibition of cancer cells.

## Figures and Tables

**Table 1:** Oxidation parameters of rapeseed oils without ant with β-sitosteryl sinapate stored under accelerated conditions.

Storage Time (Weeks)	Oxidation Parameters						
	PV * ± SD (meq O_2_/kg)	*p*-AnV * ± SD	TOTOX	K_232_ * ± SD	K_268_ * ± SD	AV * ± SD (mg NaOH/g)	FFA * ± SD (%)
**Refined Rapeseed Oil without β-Sitosteryl Sinapate**							
0	0.10 ± 0.05 ^a^	0.77 ± 0.05 ^a^	4.79	2.775 ± 0.010 ^d^	0.522 ± 0.009 ^a^	0.0487 ± 0.0012 ^a^	0.0344 ± 0.0008 ^a^
1	2.94 ± 0.23 ^b^	2.12 ± 0.08 ^b^	7.99	2.824 ± 0.013 ^e^	0.562 ± 0.006 ^c^	0.0837 ± 0.0015 ^e^	0.0591 ± 0.0010 ^e^
2	8.51 ± 0.66 ^g^	4.74 ± 0.03 ^e^	21.75	2.832 ± 0.017 ^e,f^	0.605 ± 0.012 ^d^	0.0954 ± 0.0009 ^g^	0.0674 ± 0.0007 ^g^
3	9.98 ± 0.28 ^h^	8.44 ± 0.47 ^f^	28.39	2.850 ± 0.003 ^f^	0.626 ± 0.010 ^e^	0.1110 ± 0.0006 ^h^	0.0785 ± 0.0006 ^h^
4	12.41 ± 0.28 ^i^	10.42 ± 0.28 ^g^	35.24	2.882 ± 0.006 ^g^	0.704 ± 0.012 ^f^	0.1257 ± 0.0007 ^i^	0.0888 ± 0.0005 ^i^
**Refined Rapeseed Oil with β-Sitosteryl Sinapate**							
0	2.24 ± 0.15 ^b^	0.71 ± 0.04 ^a^	5.19	2.415 ± 0.005 ^a^	0.523 ± 0.005 ^a^	0.0480 ± 0.0002 ^a^	0.0339 ± 0.0002 ^a^
1	3.56 ± 0.45 ^c^	2.27 ± 0.27 ^b^	9.40	2.430 ± 0.003 ^a,b^	0.527 ± 0.006 ^a,b^	0.0578 ± 0.0007 ^b^	0.0408 ± 0.0005 ^b^
2	4.25 ± 0.13 ^d^	2.78 ± 0.07 ^c^	11.27	2.433 ± 0.008 ^b,c^	0.531 ± 0.004 ^a,b^	0.0652 ± 0.0018 ^c^	0.0460 ± 0.0013 ^c^
3	4.84 ± 0.15 ^e^	3.52 ± 0.10 ^d^	13.20	2.438 ± 0.008 ^b,c^	0.538 ± 0.003 ^b^	0.0753 ± 0.0013 ^d^	0.0532 ± 0.0010 ^d^
4	6.00 ± 0.06 ^f^	4.54 ± 0.12 ^e^	16.54	2.448 ± 0.004 ^c^	0.540 ± 0.004 ^b^	0.0864 ± 0.0009 ^f^	0.0610 ± 0.0007 ^f^

* *n* = 3; SD—Standard Deviation; Different letters (^a–i^) within the same column indicate significant differences between oxidation parameters of the stored oils during 4 weeks (one-way ANOVA and Duncan test, *p* < 0.05).

**Table 2:** Antioxidant activity of rapeseed oils without ant with β-sitosteryl sinapate stored under accelerated conditions.

Storage Time (Weeks)	Antioxidant Activity (µmol TE/100 g)		
	ABTS * ± SD	DPPH * ± SD	FC * ± SD
**Refined Rapeseed Oil without β-Sitosteryl Sinapate**			
0	2384.8 ± 21.1 ^f^	606.0 ± 5.6 ^d^	129.4 ± 4.8 ^f,g^
1	1824.1 ± 42.4 ^d^	524.6 ± 11.9 ^c^	112.8 ± 2.6 ^d^
2	1659.9 ± 42.7 ^c^	488.9 ± 17.2 ^b^	89.6 ± 2.1 ^c^
3	1469.2 ± 17.7 ^b^	460.9 ± 5.0 ^a^	71.9 ± 3.7 ^b^
4	1346.8 ± 56.6 ^a^	452.6 ± 10.1 ^a^	59.1 ± 2.3 ^a^
**Refined Rapeseed Oil with β-Sitosteryl Sinapate**			
0	2505.6 ± 60.9 ^g^	806.2 ± 6.9 ^h^	132.3 ± 4.7 ^g^
1	2332.5 ± 79.1 ^f^	784.7 ± 7.4 ^g^	126.8 ± 2.8 ^f^
2	2116.0 ± 41.6 ^e^	765.1 ± 14.6 ^f^	120.4 ± 3.4 ^e^
3	2095.8 ± 47.8 ^e^	771.8 ± 12.3 ^f,g^	117.2 ± 3.6 ^e^
4	2119.3 ± 42.9 ^e^	748.6 ± 5.3 ^e^	117.3 ± 1.9 ^d,e^

* *n* = 5; SD—Standard Deviation; Different letters (^a–h^) within the same column indicate significant differences between antioxidant activity of the stored oils during 4 weeks (one-way ANOVA and Duncan test, *p* < 0.05).

## Data Availability

The data presented in this study are available on request from the corresponding author.
